# Secondary healing strategy for difficult wound closure in invasive vulvar cancer: a pilot case-control study

**DOI:** 10.6061/clinics/2019/e1218

**Published:** 2019-08-27

**Authors:** Altamiro Ribeiro Dias-Jr, José Maria Soares-Jr, Maria Beatriz Sartor de Faria, Maria Luiza Noqueira Dias Genta, Jesus Paula Carvalho, Edmund C Baracat

**Affiliations:** I Disciplina de Ginecologia, Departamento de Ginecologia e Obstetricia, Instituto do Cancer do Estado de Sao Paulo (ICESP), Hospital das Clinicas HCFMUSP, Faculdade de Medicina, Universidade de Sao Paulo, Sao Paulo, SP, BR; II Disciplina de Ginecologia, Departamento de Ginecologia e Obstetricia, Hospital das Clinicas HCFMUSP, Faculdade de Medicina, Universidade de Sao Paulo, Sao Paulo, SP, BR

**Keywords:** Invasive Vulvar Cancer, Radical Vulvectomy, Secondary Healing

## Abstract

**OBJECTIVES:**

Despite the number of surgical advances and innovations in techniques over time, radical vulvectomy frequently results in substantial loss of tissue that cannot be primarily closed without tension, the mobilization of surrounding tissues or even the rotation of myocutaneous flaps. The aim of this study was to evaluate the feasibility of leaving the surgical vulvar open wound for secondary healing in situations where primary closure of the vulvar wound is not possible.

**METHODS:**

This case-control pilot study analyzed 16 women with a diagnosis of squamous cell carcinoma of the vulva who first underwent inguinofemoral lymphadenectomy, 6-week sessions of chemotherapy and 25 daily sessions of radiotherapy. Afterward, excision of the vulvar lesion with free margins was performed between January 2011 and July 2017. Twelve patients underwent primary closure of the wound (control), and in 4 patients, the surgical wound was left open for secondary healing by means of a hydrofiber (case). The inclusion criteria were a) FIGO-2009 stage II up to IIIC; b) squamous cell carcinoma; and c) no evidence of pelvic or extrapelvic disease or pelvic nodal involvement. The exclusion criteria were extrapelvic disease or pelvic nodal involvement, another primary cancer, or a poor clinical condition. ClinicalTrials.gov: NCT02067052.

**RESULTS:**

The mean age of the patients at the time of the intervention was 62.1. The distribution of the stages was as follows: II, n=6 (37 %); IIIA, n=1 (6%), IIIB, n=1 (6%) and IIIC, n=8 (51%). The mean operative time was 45 minutes. The hospital stay duration was 2 days. Full vulvar healing occurred after an average of 30 days in the control group and after an average of 50 days in the case group.

**CONCLUSION:**

A secondary healing strategy may be an option for the treatment of vulvar cancer in situations of non-extensive surgical wounds when primary closure of the wound is not possible.

## INTRODUCTION

Vulvar cancer currently accounts for approximately 5% of all gynecological malignancies, and the incidence is increasing in developed countries 1. Currently, the mainstay of oncological treatment for locally advanced disease is radical vulvectomy with inguinofemoral lymphadenectomy followed by radiotherapy and/or chemotherapy. This procedure is complex and has many problems and side effects. Disruptions in wound healing are associated with impaired quality of life and may negatively impact prognosis because most of these patients need to postpone adjuvant therapies 2,3. Therefore, new approaches are necessary to improve wound healing.

Despite the number of surgical advances and innovations in techniques over time, radical vulvectomy frequently results in substantial loss of tissue that cannot be primarily closed without tension, the mobilization of surrounding tissues or even the rotation of myocutaneous flaps 4. In fact, a large wound size, tension on the suture line, surgery close to contaminated areas and comorbidities, such as diabetes and obesity, may contribute to wound breakdown 5. All of these factors must be cautiously balanced before the primary closure of the wound. The utility of leaving the operative wound deliberately open for secondary healing is not well established. This is the reason for our pilot study. This procedure does not delay oncological treatment because our patients always receive chemotherapy and radiotherapy at the site of the vulva and lymph nodes at least 40 days after lymphadenectomy, when the healing of the lymphadenectomy is complete.

The current pilot study aims to address particular situations in which primary closure of the vulvar defect is not possible and when the use of flaps or grafts is not advisable (risk of contamination, severe tissue damage or the patient's poor clinical condition). The primary objective of this pilot study was to evaluate the feasibility and operative outcomes of leaving the surgical defect open for secondary healing with the aid of a hydrofiber 6, with the outcomes including the complication rates of the patients with locally advanced vulvar cancer who were treated first with inguinofemoral lymphadenectomy and chemoradiation and then with excision of the vulvar lesion with free margins.

## METHODS

### Study design

The study was conducted at Disciplina de Ginecologia, Departamento de Obstetrícia e Ginecologia, Instituto do Câncer do Estado de São Paulo, Faculdade de Medicina da Universidade de São Paulo, Brazil. After ethical board review approval (#03427312.4.0000.0065), we conducted a pilot study with 16 cases of invasive and locally advanced vulvar cancer. All participants have provided written informed consent to participate in the study. This study was included in a large protocol that enrolled patients with advanced-stage tumors when the superficial fascia was incised, and all palpably enlarged nodes were removed and sent for frozen section analysis. If proven positive for metastatic spread, no further groin dissection was undertaken. If not, a full inguinofemoral lymphadenectomy was performed. Our protocol is included at ClinicalTrials.gov: NCT02067052.

After nodal debulking of the groin, patients received cisplatin at 40 mg/m^2^ once per week for 7 weeks throughout radiotherapy, which was performed with a dose of 45-50 Gy at 1.8 Gy/day, 5 times per week. If the lymph nodes were compromised only superficially, radiation involved only the inguinal drains and the primary lesion. This strategy aimed to decrease the vulvar lesion by reducing the morbidity of the posterior excision and to allow the rapid initiation of chemoradiation (30 to 40 days) because the complete healing of the femoral inguinal lymphadenectomy occurred at approximately 30 days, and the patients were usually discharged on the day following surgery.

Sixteen women with squamous cell carcinoma of the vulva who underwent inguinofemoral lymphadenectomy followed by chemoradiation therapy were analyzed in this study. Surgical excision of the vulvar lesion with free margins followed chemoradiation therapy. Patients were treated between January 2011 and July 2017.

Among the 16 included patients, 12 underwent primary closures of the surgical wound (controls). In four patients, the vulvar surgical wound was deliberately left open for secondary healing (cases).

Primary outcome: We evaluated whether the procedure in the hydrofiber group (case group) resulted in a similar outcome to that in the control group (primary tension-free closure) for full vulvar healing. To this end, a clinical evaluation was performed every 10 days. The potential confounders were clinical condition, age and body mass index (BMI), which may influence the results.

### Eligibility criteria

Inclusion criteria were a) FIGO-2009 stage II to IIIC; b) squamous cell carcinoma; and c) no evidence of pelvic or extrapelvic disease or pelvic nodal involvement on initial staging by imaging (CT scan and pelvic MRI). The exclusion criteria were extrapelvic disease, pelvic nodal involvement, another primary cancer or a poor clinical condition for the surgical procedure.

### Clinical variables

The following preoperative information was collected from the medical records and included: age of the patients at diagnosis, BMI, tumor size (according to clinical examination and MRI), histological type and grade of differentiation. Analyzed intraoperative (IO) data were as follows: operating time and the impossibility of primary tension-free closure. The tumor was measured using a pachometer, and data were included in the medical files. Finally, postoperative (PO) results, namely, the final pathology and presence of residual disease, the duration of the hospital stay, postsurgical complications and oncological follow-up, were documented. Additionally, we included the conventional outcome parameters used in the evaluation of a surgical outcome, namely, functional results, such as changes in urinary flow, vaginal stenosis, tropism and retraction of scars; symmetry with respect to the midline; sensory disturbances, such as pain, dysesthesia, paresthesia, and alterations in sexual function; and cosmetic aspects.

### Procedure

The decision to leave all or part of the surgical wound open for secondary healing was made intraoperatively based on the absolute impossibility of primary tension-free closure due to the extension of the vulvar defect (not larger than 4.5 cm) and/or the patient's clinical condition and associated comorbidities. After the resection of the vulvar tumor, a pathologist ensured that the resected tumor margins were free of disease. All patients were operated on by the same surgeon. The decision to close the wound was based on the evaluations of three surgeons.

After hospital discharge, prophylactic antibiotics were administered for seven days. Patients, relatives and/or responsible caregivers were extensively counseled and trained to deliver special care to the wound, including daily local hygiene and disinfection with chlorhexidine, followed by wound dressing with a hydrofiber dressing (a sterile dressing comprising sodium carboxymethylcellulose and silver that was 15x15 cm, used for infected wounds with moderate to large exudation, kept moist and aided the autolytic debridement; Aquacel Ag^TM^, ConvaTec, Princeton, NJ, USA). The dressing was changed three times per day (U$ 450.00 for the entire treatment based on the USA price). Clinical control of the wound was performed by the same examiners (the surgeon and nurse) on a weekly basis for 10 weeks.

### Visits

Control patients were followed in the same way and for the same period. Potential bias may have been introduced into our study by the timing of the evaluation, which occurred every 10 days. The wounds of some patients may have closed before the day of the evaluation.

### Statistical Methods

This is a pilot study, and a power calculation was not performed. For the statistical analysis, the mean and standard deviation of each variable was determined. We used the chi-square test to assess the relationships among the categorical variables mentioned above; *p-*values less than 0.05 were considered statistically significant. For other variables, the unpaired Student's t-test was used. All statistical analyses were conducted using the Statistical Package for the Social Sciences (SPSS, SPSS Inc., Chicago, IL, US) software, version 16.01.

## RESULTS

**Figure 1:**
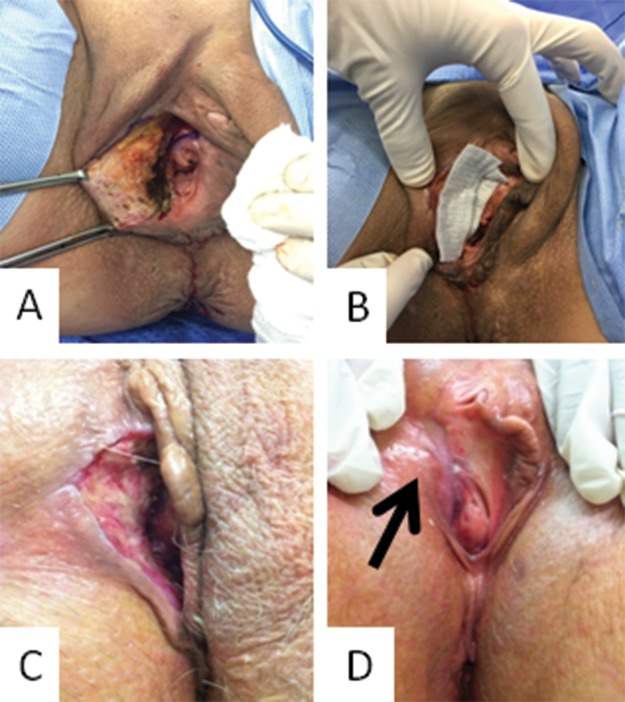
Photograph of the vulvar region of a patient with the secondary healing strategy: **A.** Vulvar size defect after resection (immediately after surgery); **B.** Curative approach with the hydrofiber; **C.** Follow-up 2 weeks after surgery; **D.** follow-up 7 weeks after surgery.

Figure 1 presents the algorithm of the study. All of the women underwent total resections of their vulvar tumors with free margins confirmed by anatomical pathological exams. Before surgery, five patients were excluded from the protocol due to poor clinical conditions (Figure 1). The mean age of the included patients at the time of the intervention was 62.1 years (39-79), while the average BMI was 27.1 kg/m^2^ (19-32.5). Histology revealed invasive squamous cell vulvar carcinoma in all cases. The distribution of the stages according to the revised FIGO 2009 staging system for vulvar cancer was as follows: II, n=6 (37%); IIIA, n=1 (6%), IIIB, n=1 (6%) and IIIC, n=8 (51%). The clinical features were similar among groups, including functional results, such as changes in urinary flow, vaginal stenosis, tropism and retraction of scars; symmetry with respect to the midline; sensory disturbances, such as pain, dysesthesia, paresthesia, and alterations in sexual function; and cosmetic aspects (Tables 1 and 2).

No deaths related to surgery occurred. The mean operative time was approximately 45 minutes, ranging from 30 to 55 minutes. The average hospital stay was 2 days (1 to 3 days). Two cases of wound infection (control group), 1 case of disease progression (case group), and 4 cases of wound breakdown (control group) were observed. Full vulvar healing occurred after an average of 29.9 days in the control group and after an average of 49 days in the case group (Figure 1A-D). The two-year survival evaluation was similar between both groups. None of the patients in the experimental group had clinical conditions for myocutaneous flaps.

## DISCUSSION

The modern concept for the treatment of advanced vulvar cancer is radical excision of the vulvar tumor and inguinofemoral lymphadenectomy, knowing that the optimal range of the free margin is 8 mm. While a microscopic margin of 2 mm seems sufficient (80% of relapses occur in patients with surgical margins less than 2 mm), less radical surgery is associated with an increased risk of relapse 7. The condition of the margins is the most important predictor of recurrence. However, this process has some problems, namely, difficulties in performing procedures such as the myocutaneous flap due to sizes, localization and/or clinical conditions, such as noncompensation diabetes and systematic arterial hypertension 8. Herein, we describe an alternative approach with a hydrofiber for these patients. The previous chemoradiation reduces the size of the lesion to obtain better conditions for vulvectomy and to close the wound. However, the side effects of this treatment (our protocol) interfere with the maintenance of a good condition for rotating or using the local dermoipodermal flaps. Therefore, the hydrofiber may be an alternative treatment for these patients to avoid uncovered wounds.

Extension of surgery, increased age and BMI, diabetes, smoking, prior radiotherapy with or without chemotherapy, and local infection are important risk factors for wound breakdown. The location of the lesion in the vulva is also a relevant element that should be taken into account because central and bilateral tumors are at increased risk for postoperative dehiscence. Normally, wound infection and breakdown following surgery become clinically evident approximately 11 days after the procedure. Up to 58% of patients undergoing vulvectomy suffer from complications related to wound healing. Consequently, the radicality of the surgery must be balanced with other risk factors to reduce morbidity but preserve oncological safety 9. Disturbances in these procedures may result in a poor prognosis for patients with vulvar cancer. Therefore, if we ameliorate wound healing, the oncological prognosis can also be improved.

The prevention and treatment of wound infection and wound breakdown in the vulva follow the general principles after surgery to the groin. Flaps, grafts and hyperbaric oxygen therapy are additional strategies for prevention 10,11. When the use of these strategies is not possible, such as in patients with major comorbidities, in patients with prior chemoradiation or in situations in which the surgical wounds are not too extensive, the strategy of leaving the surgical wound open for secondary healing enhanced by the use of a hydrofiber dressing may be taken into consideration. The mechanism involves decreasing the risk of bacterial colonization and stimulating granulation tissue. These steps also help the operative wound stay dry, speeding re-epithelialization 6. In our study, we adopted the strategy of leaving the surgical wound open for secondary healing in four patients. The healing time was faster, but cicatrization occurred. The delay to adjuvant therapy arising from the time for cicatrization was not relevant for prognosis because all patients had received chemoradiation. Therefore, the oncological treatment of the patients was not disrupted.

Finally, our study has some limitations because it is a pilot study with a small number of patients. Keeping patients from obtaining any wound protection if the wound can be closed is unethical because of the potential for infection. Therefore, we did not include a control without the hydrofiber. Additionally, the choice of closing the wound or using the hydrofiber was based on the local conditions after the chemoradiation treatment. The potential bias of our study involves the evaluation that was performed every 10 days. The wounds of some of the patients may have closed before the day of the evaluation. The strength of our study is the possibility of closing the wound with a hydrofiber dressing.

## CONCLUSION

A secondary healing strategy may be an option for the treatment of vulvar cancer in situations of nonextensive surgical wounds when primary closure of the wound is not possible. Further studies are needed to evaluate the use of the hydrofiber in the routine clinical practice of the surgical treatment of patients with vulvar cancer.

## AUTHOR CONTRIBUTIONS

Dias-Jr AR provided substantial contributions to the conception and design of the study, acquisition of the data, analysis and interpretation of data, was involved in drafting the manuscript and revising it critically for important intellectual content, provided final approval of the version to be published, and agrees to be accountable for all aspects of the work in terms of ensuring that questions related to the accuracy or integrity of any part of the work are appropriately investigated and resolved. Soares-Jr JM, de Faria MBS, Genta MLND were involved in drafting the manuscript and revising it critically for important intellectual content, provided final approval of the version to be published, and agreed to be accountable for all aspects of the work in terms of ensuring that questions related to the accuracy or integrity of any part of the work are appropriately investigated and resolved. Carvalho JP provided substantial contributions to the conception and design of the study, acquisition of the data, analysis and interpretation of the data, provided final approval of the version to be published, and agreed to be accountable for all aspects of the work in terms of ensuring that questions related to the accuracy or integrity of any part of the work are appropriately investigated and resolved. Baracat EC was involved in drafting the manuscript and revising it critically for important intellectual content, provided final approval of the version to be published, and agreed to be accountable for all aspects of the work in ensuring that questions related to the accuracy or integrity of any part of the work are appropriately investigated and resolved.

## Figures and Tables

**Table 1:** Clinical features of the patients.

Stage (FIGO)	Age (years)^a^	BMI^b^ (kg/m^2^)^b^	Tumor size (mm)*,^c^	Length of wound (mm)**,^d^	Width of wound (mm)**,^e^	Comorbidity^f^
Experimental group						
IIB	65	17.7	60	42	39	Hypothyroidism
IIIC	46	26.1	50	41	31	Hypertension***
IIIC	59	24.5	55	40	33	Hypertension***
IIIC	76	24.8	45	42	20	Hypertension***/Diabetes Mellitus
Control group						
IIB	75	25.3	41	31	33	Hypertension***
IIB	67	25.1	60	38	32	None
IIIC	39	33.3	80	39	31	Diabetes Mellitus
IIIA	79	27.5	45	28	22	Hypertension***
IIB	56	23	40	39	33	None
IIIC	50	27	41	38	30	Hypertension***
IIIC	43	34.2	50	31	32	Glaucoma
IIB	42	27.7	60	31	29	Leukemia
IIIC	77	24.5	60	39	31	Hypertension***/Diabetes Mellitus
IIB	70	17.3	41	31	35	Hypertension***/Diabetes Mellitus/CKI
IIB	72	27.7	41	39	37	Hypertension***
IIIC	77	22.5	50	39	38	Hypertension***

* Maximum diameter before chemoradiation therapy;

** after chemoradiation therapy;

*** systemic arterial hypertension;

a *p*=0.93 (experimental group versus control group, using unpaired Student’s t-test);

b *p*= 0.26 (experimental group versus control group, using unpaired Student’s t-test);

c *p*=0.79 experimental group versus control group, using unpaired Student’s t-test);

d *p*=0.23 (Experimental group versus control group, using unpaired Student’s t-test);

e *p*=0.79 (experimental group versus control group, using unpaired Student’s t-test);

f *p*=0.28 comparing the presence of systemic arterial hypertension between both groups (Fisher’s exact test).

**Table 2:** Functional results after surgical procedures.

	Experimental group (n=4)	Control group (n=12)	RR (95% confidence interval)	p*
Urinary flow abnormal	1	5	0.56 (0.074 to 4.21)	0.98
Pain	2	3	2.20 (0.42 to 11.47)	0.55
Dysesthesia	1	6	0.43 (0.06 to 3.29)	0.59
Paresthesia	3	8	1.36 (0.18 to 10.09)	0.99
Alteration in sexual function	3	9	1.00 (0.14 to 7.10)	1.00
No sexual activity	2	5	1.29 (0.24 to 6.99)	1.00
Vaginal stenosis	1	8	0.26 (0.03 to 1.99)	0.26
Retraction of scars	1	6	0.43 (0.06 to 3.29)	0.58
Tropism disturbance	0	1	-	-
Symmetry with respect to the midline	3	5	3.00 (0.39 to 23.08)	0.57

* Fisher’s exact test; RR = Relative Risk.

## References

[1] Woelber L, Trillsch F, Kock L, Grimm D, Petersen C, Choschzick M (2013). Management of patients with vulvar cancer: a perspective review according to tumour stage. Ther Adv Med Oncol.

[2] Wills A, Obermair A (2013). A review of complications associated with the surgical treatment of vulvar cancer. Gynecol Oncol.

[3] Magrina JF, Gonzalez-Bosquet J, Weaver AL, Gaffey TA, Webb MJ, Podratz KC (1998). Primary squamous cell cancer of the vulva: radical versus modified radical vulvar surgery. Gynecol Oncol.

[4] Walker KF, Day H, Abu J, Nunns D, Williamson K, Duncan T (2011). Do surgical techniques used in groin lymphadenectomy for vulval cancer affect morbidity rates?. Int J Gynecol Cancer.

[5] De Hullu JA, Hollema H, Lolkema S, Boezen M, Boonstra H, Burger MP (2002). Vulvar carcinoma. The price of less radical surgery. Cancer.

[6] Caruso DM, Foster KN, Hermans MH, Rick C (2004). Aquacel Ag in the management of partial-thickness burns: results of a clinical trial. J Burn Care Rehabil.

[7] Arvas M, Kahramanoglu I, Bese T, Turan H, Sozen I, Ilvan S (2018). The Role of Pathological Margin Distance and Prognostic Factors After Primary Surgery in Squamous Cell Carcinoma of the Vulva. Int J Gynecol Cancer.

[8] Leminen A, Forss M, Paavonen J (2000). Wound complications in patients with carcinoma of the vulva. Comparison between radical and modified vulvectomies. Eur J Obstet Gynecol Reprod Biol.

[9] Hinten F, van den Einden LC, Hendriks JC, van der Zee AG, Bulten J, Massuger L (2011). Risk factors for short- and long-term complications after groin surgery in vulvar cancer. Br J Cancer.

[10] Schimp VL, Worley C, Brunello S, Levenback CC, Wolf JK, Sun CC (2004). Vacuum-assisted closure in the treatment of gynecologic oncology wound failures. Gynecol Oncol.

[11] Thackham JA, McElwain DL, Long RJ (2008). The use of hyperbaric oxygen therapy to treat chronic wounds: A review. Wound Repair Regen.

